# The Species Dilemma of Northeast Indian Mahseer (Actinopterygii: Cyprinidae): DNA Barcoding in Clarifying the Riddle

**DOI:** 10.1371/journal.pone.0053704

**Published:** 2013-01-16

**Authors:** Boni A. Laskar, Maloyjo J. Bhattacharjee, Bishal Dhar, Pradosh Mahadani, Shantanu Kundu, Sankar K. Ghosh

**Affiliations:** Department of Biotechnology, Assam University, Silchar, Assam, India; Ecole Normale Supérieure de Lyon, France

## Abstract

**Background:**

The taxonomic validity of Northeast Indian endemic Mahseer species, *Tor progeneius* and *Neolissochilus hexastichus*, has been argued repeatedly. This is mainly due to disagreements in recognizing the species based on morphological characters. Consequently, both the species have been concealed for many decades. DNA barcoding has become a promising and an independent technique for accurate species level identification. Therefore, utilization of such technique in association with the traditional morphotaxonomic description can resolve the species dilemma of this important group of sport fishes.

**Methodology/Principal Findings:**

Altogether, 28 mahseer specimens including paratypes were studied from different locations in Northeast India, and 24 morphometric characters were measured invariably. The Principal Component Analysis with morphometric data revealed five distinct groups of sample that were taxonomically categorized into 4 species, viz., *Tor putitora*, *T. progeneius*, *Neolissochilus hexagonolepis* and *N. hexastichus*. Analysis with a dataset of 76 DNA barcode sequences of different mahseer species exhibited that the queries of *T. putitora* and *N. hexagonolepis* clustered cohesively with the respective conspecific database sequences maintaining 0.8% maximum K2P divergence. The closest congeneric divergence was 3 times higher than the mean conspecific divergence and was considered as barcode gap. The maximum divergence among the samples of *T. progeneius* and *T. putitora* was 0.8% that was much below the barcode gap, indicating them being synonymous. The query sequences of *N. hexastichus* invariably formed a discrete and a congeneric clade with the database sequences and maintained the interspecific divergence that supported its distinct species status. Notably, *N. hexastichus* was encountered in a single site and seemed to be under threat.

**Conclusion:**

This study substantiated the identification of *N. hexastichus* to be a true species, and tentatively regarded *T. progeneius* to be a synonym of *T. putitora*. It would guide the conservationists to initiate priority conservation of *N. hexastichus* and *T. putitora*.

## Introduction

The term ‘mahseer’ refers to a group of freshwater cyprinid fishes easily distinguishable by relatively larger size of scales on their body compared to the other cyprinid fishes [1], [2]. The members of mahseer belong to two genera, viz., *Tor* and *Neolissochilus*. These two genera are distinguished by the presence of a continuous labial groove in *Tor* but interrupted in *Neolissochilus,* and 10–14 gill rakers on the lower arm of first gill arch in the former and 6–9 in the latter [3], [4]. They inhabit in the mountain streams and distributed in the range from Pakistan throughout Southern Asia to Southeast Asia up to the Malay Peninsula and the larger Indonesian islands across Sumatra, Borneo and Java [5], [6]. However, species composition within each genus varies in different locations, like Southeast Asian species are different from Southern Asian species. Furthermore, within India, many species of mahseer are discontinuously distributed and mostly endemic in the South, Central and Northeast India. Among the mahseer of the Indian subcontinent, *Tor putitora* is widely distributed in Pakistan, India, Nepal and Bhutan; while *Neolissochilus hexagonolepis* is distributed in Nepal, Bhutan, North India and Northeast (NE) India [7], [8]. A few studies suggest that the angling of mahseer provides superlative thrills than any other sport fishes except European Salmon [9], [10]. They are highly sought-after because of great attraction to recreational anglers and are important components of the Angling-tourism pursuit [11]. In developing countries, there are many instances where the tourism industry has added recreational fishing to their attractions [12]. Owing to the growing value, the mahseer has become popular and considered as a cultural icon of diverse economic, recreation, and conservation standpoint in rivers of eleven Asian nations [13]. Above all, the mahseer is an integral component of the aquatic ecosystem, serves as an important indicator of its health and supports the livelihood of many rural and indigenous ethnic groups in Asia [14]. However, the important mahseer fishes are threatened in the NE India as well as other distribution areas due to the growing harvest pressure as well as anthropogenic effects [15], [16]. The two most threatened species, viz., *Tor putitora* and *Neolissochilus hexagonolepis* are regarded as the flagship species in NE India (http://www.nbfgr.res.in/). The conservation of mahseer has been hampered because the taxonomy of mahseer is most confusing due to the morphological variations they exhibit [17] that poised the understanding of actual species composition, distribution, autecology and biology at large.

Historically, with the pioneering work of Hamilton-Buchanan (1822) [1], many new descriptions of different species of mahseer have been proposed from Indian waters by distinguished naturalists. McClelland (1839) [18] recorded 4 new species from NE India, viz., *Tor progeneius, T. macrocephalus, Neolissochilus hexagonolepis* and *N*. *hexastichus*. McClelland, however, admitted difficulty in identifying Hamilton’s *Cyprinus* (now *Tor*) *putitora* and particularly emphasized on a large cellular appendage to the apex of the lower jaw for *T. progeneius*, and the color gray on the back and reddish yellow on rest of the body for *N. hexastichus* [18]. The taxonomy of *T. progeneius* had long been in doubtful status, and it has been considered as a junior synonym of *T. putitora* [19]. Sen and Jayaram (1982) [20] characterized *T. progeneius* and elucidated with some new characteristics. Later, Rainboth (1985) [3] noted that *T. progeneius* is confusing to be classified whether within the genus *Neolissochilus* or *Tor*. It was further noted that most of the McClelland’s type specimens were misplaced and some constituted curatorial nightmare [3]. Yet, McClelland’s descriptions of two distinct species, viz., *Neolissochilus hexagonolepis* and *Tor progeneius* are recognized to be valid; while *T. macrocephalus* and *N. hexastichus* have been considered to be not valid rather the former was synonymized with *T. putitora* and the latter with *T. tor* [5], [21].

Thus, the traditional taxonomy of mahseer in NE India has been facing several problems due to (1) lack of morphometric details in original description, (2) presence of very few holotypes of mahseer species, (3) indiscernible morphological nuances in them, and (4) disagreements in recognizing specific morphological characters. Consequently, the taxonomy of a few mahseer species has been extremely chaotic and described severally [2], [4], [5], [20], [21], [22], [23]. The mahseer species composition in the region is poorly understood and the identification of two species, viz., *T. progeneius* and *N. hexastichus*, has been difficult due to inconsistent taxonomic descriptions. Therefore, species level identification of mahseer is needed to be strengthened to facilitate the autecological study of mahseer and to develop conservation strategy for sustainable utilization in recreational fishing based tourism. Genomic approaches of taxon diagnosis have been found to be resourceful to aid traditional taxonomy [24], [25]. In this context, the mitochondrial genome is a better target than nuclear genome because it evolved faster and can thus give more information to discriminate close species.Lately, a partial fragment of mitochondrial cytochrome oxidase c subunit I (COI) gene has been proposed to be sufficient singly to differentiate all, or at least the vast majority of animal species [26]. As such, this partial locus (COI) has been extensively tested for its efficacy in fish species identification and recognized as a unique marker of species identification with high confidence and called as “DNA barcode” [27], [28], [29]. The concept of DNA barcode based species identification is easy, rapid and accurate for being sequencing and web based; as such it has gained great attention worldwide [30], [31], [32]. Recently, the catfish diversity in NE India has been re-evaluated through DNA barcoding [33]. Therefore, morphological and DNA barcode data in combination can help to resolve the species dilemma of Northeast Indian mahseer, particularly *T*. *progeneius* and *N. hexastichus,* for effective conservation and management of the species.

## Materials and Methods

### Sample Collection

**Figure 1:**
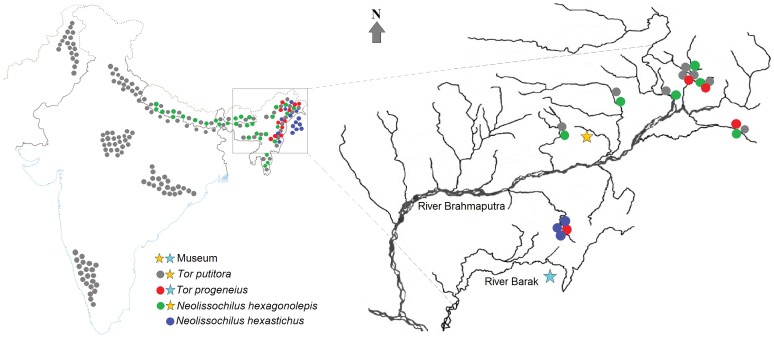
Map of the study site showing the known distribution of the studied species and the collection sites in different river drainages. The figure shows that the Northeastern region of India is drained mostly by River Brahmaputra and partly by River Barak. The studied specimens were collected from the drainages of River Brahmaputra. The topography of the region restricts the convergence of Southeast Asian fish composition with this region.

Fish specimens belonging to the group mahseer in the range of sub-adult to adult size were collected through participatory sampling with the marginal fishers engaged in commercial fishing. The specimens were from various locations in the hills and foothills across the Northeast India, particularly in the drainages of River Brahmaputra (Figure 1). The method of sample collection was approved by the Ministry of Science and Technology, Department of Biotechnology, Government of India (vide No. BT/HRD/01/002/2007). Some known voucher specimens within the genera *Tor* and *Neolissochilus* were examined from the Museum of Biodiversity in Rajiv Gandhi University, Arunachal Pradesh (voucher numbers are given in Table 1). The morphometrics of previously identified specimens from collection of *T. putitora* and *T. progeneius*, as well as the type specimens of *T. putitora* and *N. hexagonolepis* were included in the analysis. The type specimens of *T. progeneius* and *N. hexastichus* are not available in the museum. In lieu of examining type specimens of *T. progeneius* a small review on the existing contradictions among the taxonomists regarding the taxonomic descriptions and opinions on the status of the species is given in Supporting Information S1. Concerning the identification of *T. progeneius* and *N. hexastichus*, the original descriptions were emphasized. A total of 19 fresh specimens belonging to 4 species were studied in association with 5 paratypes and 4 previously collected specimens. Muscle tissue samples were invariably collected aseptically from behind of dorsal fin of the fresh specimens and taken in 500 µL of TES buffer (50 mM Tris HCl, 25 mM EDTA and 150 mM NaCl). The whole body specimens are preserved and stored at the Department of Biotechnology of Assam (Central) University, Silchar, Assam, India, for frequent examination and record of vouchers (vouchers’ details are provided in Table S1).

**Table 1:** Morphological grouping of the studied organisms along with the corresponding codes.

Group	Nomenclature in practice	Sequence accession number used in molecular analysis/catalogue number of paratypes in museum	Sample code used in morphological analysis (PCA)
N_2_	*Neolissochilus hexastichus*	SGBL-BMF35	A
		JX127237	B
		JX127239	C
		JX127235	D
		JX127236	E
		JX127238	F
		SGBL-BMF36	*
N_1_	*N. hexagonolepis*	JX127232	G
		JX127234	H
		JX127231	I
		JX127233	*
		** RGUMF-0036	V
		** RGUMF-0037	W
		** RGUMF-0038	X
T_2_	*Tor progeneius*	JX127229	J
		***	K
		JX127228	L
		***	M
		JX127230	N
T_3_	*T. putitora*	** RGUMF-0034	Aa
		JX127240	O
		JX127224	P
		JX127241	Q
		JX127242	U
T_1_	*T. putitora*	JX127227	R
		JX127226	T
		JX127225	S
		** RGUMF-0035	Y
		***	Z
		***	Ab

The grouping was done based on scatter plot from Principal Component Analysis (PCA) as well as following the authoritative taxonomic keys. Sequence accession numbers in GenBank are used in the presentation of molecular analysis and the sample codes in PCA. Alphabetic sample codes replacing the full name of organisms are ascribed for ease of presentation those however clearly mentioned in Table S2.

* big specimen from market whose morphometric not done.

** paratypes from museum preserved in formaline whose sequencing not done.

*** previously identified specimens preserved in formaline whose sequencing not done.

### Taxonomic Identification and Nomenclature

Specimens were categorized systematically based on the taxonomic characters available from the original description as well as subsequent re-descriptions and taxonomic reviews. Altogether 24 morphometric variables along with 6 important meristic counts were measured following standard literatures [23], [34] (Figure S1) and the measurements were recorded using digital slide caliper (0.01 mm). The morphological characters those are non-quantitative yet taxonomically relevant, e.g. color pattern on the body and fins, presence or absence of tubercles, appearance and diagonal shape of mouth, etc. were also recorded from all the specimens. The measurements were taken at least three times independently and mode of each parameter was finally considered to minimize the error. The samples were designated into the respective species as per the authoritative taxonomic keys [4], [20], [23] and the species nomenclature was adopted as per the updated catalogue [8].

### PCR Assay and Purification

DNA was extracted with standardized Phenol-Chloroform-Isoamyl alcohol method [35]. COI gene fragment (∼655 bp) was amplified using the set of published primers: FishF1-5′TCAACCAACCACAAAGACATTGGCAC 3′ and FishR1-5′TAGACTTCTGGGTGGCCAAAGAATCA 3′[27]. The amplification was performed in 25 µl reaction mixture of 1X PCR buffer, 2 mM MgCl_2_, 10 pmol of each primer, 0.25 mM of each dNTPs, 0.25 U high-fidelity polymerase and 100 ng of DNA template. PCR conditions were: initial denaturation at 94°C (2 minutes) followed by 30 cycles at 94°C (45 seconds), 50°C (45 seconds) and 72°C (1 minute), and a final elongation at 72°C (8 minutes). The PCR-amplified products were checked in 1% agarose gels containing ethidium bromide (10 mg/ml) and the single uniform band was then purified using QIAquick^R^ Gel extraction kit (QIAGEN, USA). The amplicons were bi-directionally sequenced in an automated DNA sequencer (ABI 3500, Applied Biosystems Inc., CA, USA).

### Sequence Quality Control Measures

Two chromatograms that represent sequences of both the DNA strands were obtained for each sample. The PCR amplified products as well as their corresponding DNA sequences were larger than 600 bp that assured the sequences being not Numts as the limit of Numt hardly reaches 600 bp [36]. The noisy sequences were trimmed at both end and greater than 2% ambiguous bases were discarded, using quality value of >40 for bidirectional reads. BLASTN program was used to compare the sequences retrieved from the two chromatograms [37], and the fragment showing 100% alignment with no gap or indel (insertion/deletions) was selected. In some cases of discrepancy, both the sequences were reviewed and quality value of the sequences were considered to determine the most likely nucleotide using the software SeqScanner Version 1.0 (Applied Biosystems Inc., CA, USA). The selected fragments of the sequence were aligned using ClustalX software [38]. Finally, each of the sequences was compared in NCBI through BLASTN to examine the complete alignment with the partial coding sequence of fish mitochondrial COI gene. The sequences were translated using the online software ORF finder (http://www.ncbi.nlm.nih.gov/gorf/gorf.html) and aligned through BLASTP to examine whether the partial amino acid codes were coherent with the fish mitochondrial COI gene frame and without any stop codon. In this way, the generated sequences were confirmed to be the fragments of mitochondrial COI gene. All the analyzed sequences were then deposited in GenBank (details of accession numbers are given in Table S1). The sequences were also submitted in a FISH-BOL project entitled “DNA barcoding of Mahseer fishes from Northeast India” and the code name ‘MFISH’.

### Data Analysis

#### Morphometry

Principal Component Analysis (PCA), a multivariate statistical procedure commonly used to reveal patterns in measured correlated variables, was used to differentiate the samples into possible groups and any variation among the samples of same species and the paratypes. The morphometric measurements were transformed into percentage of the total body length to develop the relative data of each variable for the samples of different size and species. The analysis was performed using PAST version 2.17 b (http://folk.uio.no/ohammer/past). The PCA output is presented as scatter plot showing the groups of the samples with designated codes.

#### COI sequence data analysis

The sampled specimens were invariably sequenced and their congeneric sequences were acquired from the databases (GenBank and BOLD) to examine the level of intraspecific variation. Most of the database sequences lack geographical information yet they were assumed to be at least from distant locations. The analysis was based on a total data set of 76 COI barcode sequences of mahseer containing 21 *denovo* sequences and 55 database sequences. Additionally, 2 sequences of *Hypsibarbus wetmorei* and 3 sequences of *Puntius sarana* were acquired from GenBank to represent the out-group in the study. Geographical information and GenBank accession numbers of the developed as well as acquired sequences are given in Table S1. The calculation of Kimura 2-parameter (K2P) congeneric and conspecific distance [39] as well as phylogenetic analysis through Neighbor Joining (NJ) method were performed using MEGA Version 5.1 [40]. The tree topology obtained through NJ method was double-checked by Maximum Likelihood (using MEGA Version 5.1) and Bayesian approach (using MrBayes 3.2.0) [41].

## Results

### Morphological Characteristics

**Figure 2:**
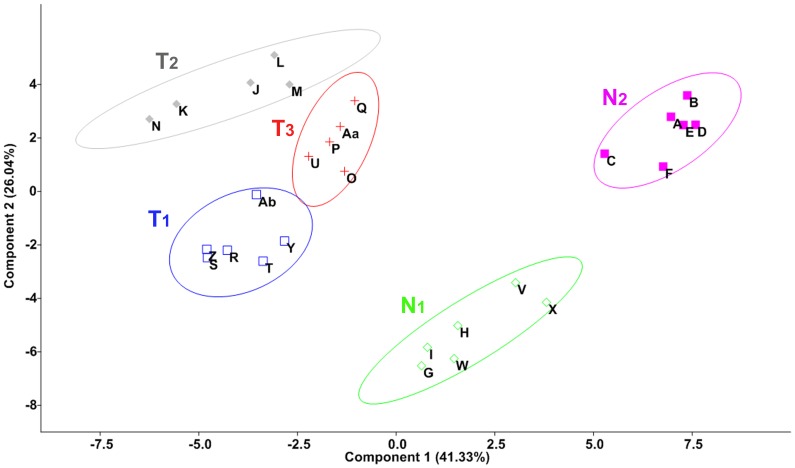
Principal Component Analysis (PCA) on 24 morphometric variables of the study samples including paratypes. The clusters of samples obtained from PCA were assigned to respective taxa based on meristic counts as well as non-quantitative characters of samples following authoritative taxonomic keys. The groups are like T_1_, T_2_ and T_3_ comprising *Tor* congener, and N_1_ and N_2_ comprising *Neolissochilus* congener.

The PCA yielded 24 components which correspond to the 24 morphometric measurements. Projection of the morphometric data of studied mahseer species on first 2 principal axes showed the separation of the samples into 5 groups at 75% concentration ellipse level (Figure 2). The first 2 principal components contributed to 67.37% of total variance (PC1 = 41.33% and PC2 = 26.04%) (Table 2). The third, fourth and fifth components contributed to 8.57%, 4.93% and 3.55%, respectively, but did not improve the separation of the samples. These 5 groups were categorized into 2 broad groups and each corresponds to a genus, as per the authoritative taxonomic keys. The meristic count of the samples is presented in Table 3 which depicts a prominent difference in number of gill rakers on the lower arm of first arch between the two genera. The rakers were 8–9 in *Neolissochilus* and 13–14 in *Tor*. The other meristics were almost similar in all the samples. In the PCA scatter plot, the samples within the genus *Neolissochilus* further formed two distinct groups, one of which grouped with the paratypes of *N. hexagonolepis* but the other group stood distant indicating both the groups belonging to different species. The samples within the genus *Tor* appeared to be in a single but very stretched out group indicating a wide range of variation. In this group, some samples formed two slightly distant groups, yet each of the groups assembled with at least one of the paratypes of *T. putitora* while the rest few samples formed a slightly separate group and remained away from the paratypes. The non-quantitative characters of samples within *Tor* and the prevailing taxonomic descriptions suggested two possible species name. The groups of samples appeared in PCA were designated as T_1_, T_2_ and T_3_ comprising *Tor* congener, and N_1_ and N_2_ comprising *Neolissochilus* congener. The constituent samples within each group were given the alphabetic sample code (Table 1), like S, R, T, Y, Z and Ab fall within T_1_; J, K, L, M, and N fall within T_2_; Aa, O, P, Q and U fall within T_3_; G, H, I, V, W and X fall within N_1_; and A, B, C, D, E and F fall within N_2_. The meristic counts and morphometric data are given in Table 3 and supplementary Table S2 respectively.

**Table 2:** Summary of PCA on 24 morphometric measurements of 28 samples within 4 species.

	PC 1	PC 2
% variance	41.336	26.04
Eigen value	19.4715	12.2662
Variable	Loadings	
SL	−0.3807	−0.1508
PrDL	−0.1599	0.2042
PoDL	0.1929	−0.1689
HtCF	0.298	0.1092
HL	−0.2624	0.3204
HtPF	0.1026	0.09338
HtDF	0.2399	0.3027
HtAF	0.1577	0.1663
HtDS	0.1129	0.1021
DP&V	−0.1125	−0.4824
LnCP	−0.1836	−0.1738
BDdf	0.4392	−0.1007
HDop	0.2464	−0.1186
HDe	0.1665	−0.03199
BWdf	0.1187	−0.1101
HWe	0.1814	0.00083
SnL	−0.1136	0.1563
ED	0.0623	0.07016
LnLF	−0.182	0.363
LHtCP	0.1185	−0.00832
HtVF	0.1842	0.07278
DVF&AF	−0.08024	−0.4201
LnBDF	0.2209	−0.09064
LnBAF	0.03401	−0.05084

Proportion of variance, Eigen values and coefficients (loadings) of the first two principal components (PC1 and PC2) for the % total length of the morphometrics of studied mahseer species.

**Table 3:** Important meristic counts of three specimens in each species.

Organism name (Species)	Replicates	Parameters					
		Gill rakers on first arch (upper arm+lower arm)	Scales on lateral line	Dorsal fin rays	Ventral fin rays	Pectoral fin rays	Anal fin rays
*Tor putitora*	a	2+14	25	9+ii	8+i	15+i	6+i
	b	2+14	25	9+ii	8+i	15+i	6+i
	c	2+14	25	9+ii	8+i	15+i	6+i
*Tor progeneius*	a	2+14	26	9+ii	8+i	15+i	6+i
	b	2+13	26	9+ii	8+i	15+i	6+ii
	c	2+14	26	9+ii	8+i	15+i	6+i
*Neolissochilus hexagonolepis*	a	2+8	27	9+ii	8+i	14+i	6+i
	b	2+8	27	9+ii	8+i	14+i	6+i
	c	2+8	27	9+ii	8+i	14+i	6+i
*Neolissochilus hexastichus*	a	2+9	24	10+ii	7+i	14+i	7+i
	b	2+9	24	10+ii	7+ii	14+i	7+i
	c	2+9	24	10+ii	8+i	14+i	7+i

The table shows that the number of gill rakers on the lower arm of first arch is a very important distinguishing character between the two genera. This character is very easily identifiable and based on this character the first hand classification of mahseer in to respective genera can be easily done.

• Lowercase roman numerals are used to denote the simple rays in fin ray count.

### *Tor* Congener

The first taxonomic key to differentiate species within *Tor* is based on the relative head length to the body depth. In this study, all *Tor* congener possessed slightly longer head than body depth. There was no stringent variation in meristic counts among the *Tor* congener (Table 3); and both T_1_ and T_2_ samples were similar in most of the other taxonomic features (Table S2). But, T_3_ differed from T_1_ and T_2_; firstly on having long (up to the margin of maxilla) mental lobe (also called lower labial flap) vs. short/absent, and secondly on having longer upper jaw and with skin like flap extending behind upper lip vs. both the jaws equal and upper lip without a flap. The mental lobe length was 4.29% to 5.91% of total length in T_3_ samples vs. 1.47% to 1.99% in T_1_ and T_2_ samples. The longer upper jaw in T_3_ samples correspondingly shared to greater head length and snout length than in T_1_ and T_2_ samples. It appeared that the presence of both upper and lower lips as being relatively more fleshy and the mental lobe being prominent and long in all the samples of T_3_ differentiate them from T_1_ and T_2_ samples. The observed morphological features of all the samples within T_1_ and T_2_ bear close affinity with the described features of *Tor putitora*. Therefore, despite minor differences, T_1_ and T_2_ samples were considered to be belonging to the same species and named accordingly. The particular lip character in T_3_ samples resemble with the original descriptions of *Tor progeneius*. It was observed that the three groups though bear minor variation in morphometrics but they are not discernible except the particular differentiating features of *Tor progeneius* that appeared to be unique and very much noticeable. Thus, T_3_, T_1_ and T_2_ samples are tentative considered as morphs and the former is designated as long mental lobed while the latter 2 as short mental lobed.

### Neolissochilus Congener

The N_1_ samples were distinguished from N_2_ due to absence of mental lobe vs. prominent, and interrupted groove behind the lower lip vs. continuous groove. Both these features of N_2_ samples resembled with the *Tor* congener. But, the gill rakers in them were 9 vs. 13–14 in *Tor* congener. Tubercles were mostly present on the cheeks in N_1_ but entirely absent in N_2_. Mouth smoothly rounded in N_2_ vs. truncate in N_1_, edge of lower jaw blunts in N_2_ vs. sharp in N_1_. The color of the back, bases of caudal and dorsal as well as the upper part of the head in N_2_ samples was greenish gray, reddish yellow on rest of the body, and the tips of the fins red. These observed features in N_2_ samples have been originally emphasized to describe *N. hexastichus* as a distinct species. Thus, the N_2_ samples were named as *N. hexastichus* according to the authoritative descriptions. The morphometrics of N_1_ samples were mostly similar to both *N. hexagonolepis* and *N. stracheyi*. The N_1_ samples were uniquely identified to be *N. hexagonolepis*, based on color pattern having scales coppery colored with a tinge of red above lateral line and fins deep slate paling towards their margins. As per the prevailing taxonomic description, *N. hexagonolepis* is different from *N. stracheyi* due to the absence of a lateral black stripe as in N_1_ samples. Thus, following the taxonomic keys, the N_1_ samples were named as *N. hexagonolepis*.

### DNA Barcoding Analyses

The K2P divergence matrix of the dataset (as shown in Table S3) revealed that the congener of *Tor* maintained divergences in the range of 3.5% to 7.4% with the congener of *Neolissochilus*. The maximum K2P divergence among T_1_ and T_2_ samples was 0.2% and the comparison of both T_1_ and T_2_ samples with T_3_ samples also revealed a maximum K2P divergence of 0.2%. The maximum divergence of all the samples belonging to T_1_, T_2_ and T_3_ with the closest database sequences of *Tor putitora* was 0.8%. The divergence matrix suggested that all samples of T_1_, T_2_ and T_3_ are conspecific of *T. putitora* in the absence of any database sequence of *T. progeneius*. Therefore, COI gene sequences of these 3 groups were submitted to both GenBank and BOLD under the putative species *T. putitora*. The maximum divergence within N_1_ samples was 0.6% while their divergences with the conspecific database sequences were in the range of 0.4% to 0.8%. The divergence matrix suggested that N_1_ samples are conspecific of *Neolissochilus hexagonolepis*. The within group divergences of N_2_ samples were in the high range up to 0.9% possibly due to a particular sequence. Excluding the particular sequence (accession number JX127239), the within group divergence of N_2_ samples remained nil in the absence of any conspecific sequence in the database.

The averages of conspecific and congeneric divergences were determined from the matrix to be 0.5% ±0.2% and 2.8% ±0.7% respectively. In the dataset, the minimum distance between the closest species (closest congener) was 1.5%. Therefore, the closest congeneric divergence among mahseer species was calculated to be 3 times higher than the mean conspecific divergence, which is called as the ‘barcode gap’. Based on the barcode gap, T_1_, T_2_ and T_3_ samples were found to be conspecific with *T. putitora*; N_1_ samples were conspecific with *N*. *hexagonolepis*; and N_2_ samples including JX127239 were discrete in the absence of any database sequence of *N*. *hexastichus*.

**Figure 3:**
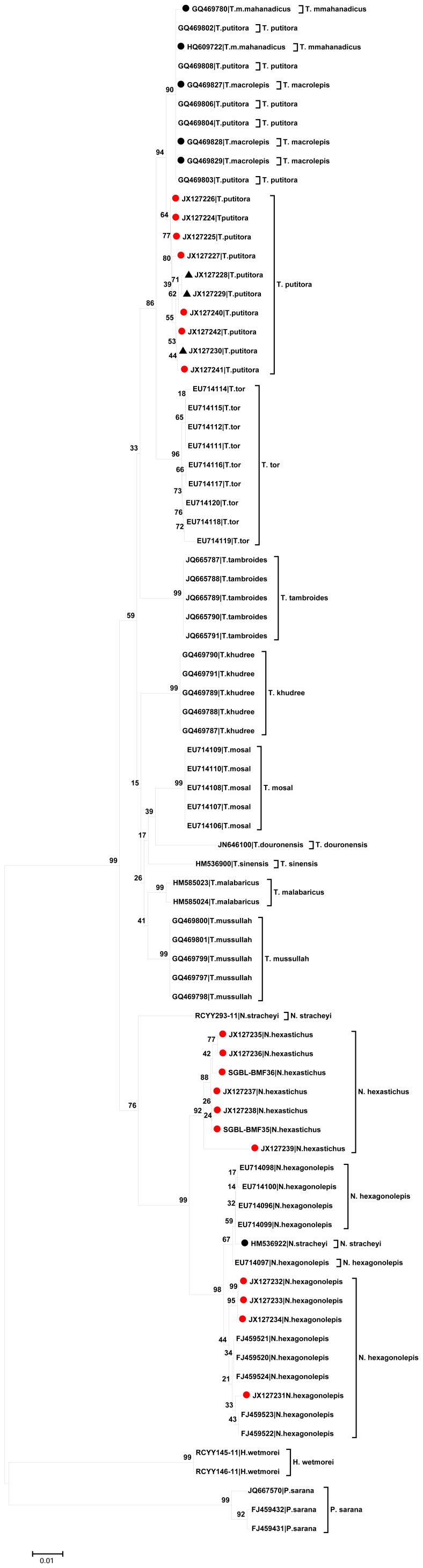
Neighbor Joining (NJ) tree developed using K2P distance among 76 COI sequences of mahseer. In most cases the studied samples (marked as red dots) showed cohesive clustering with their conspecific database sequences. The samples morphologically identified as *Tor progeneius* (accession numbers JX127229, JX127230 and JX127228) clustered conspecific with developed sequences as well as database sequences of *T. putitora.* All samples of *N. hexastichus* clustered cohesive as the same species and distinct from *N. hexagonolepis* sequences. Few database sequences of 3 species revealed aberrant clustering {*Neolissochilus stracheyi* (accession number HM536922), *Tor mosal mahanadicus* (accession numbers HQ609722, GQ469780), *Tor macrolepis* (accession numbers GQ469827-29)}. • The numbers at the nodes are bootstrap values based on 1000 replications. • The specimens’ GenBank accession number and species name are shown for each taxon. • Red dots and black triangles correspond to the sequences developed in this study. Black triangles also correspond to the sequences of samples, although morphologically identified as *Tor progeneius* but were found conspecific with *Tor putitora* based on COI sequence data analysis. Black dots correspond to the cases of abnormal clustering.

The NJ as well as Maximum Likelihood (ML) and Bayesian tree based cluster revealed that the congener of *Tor* and *Neolissochilus* formed two related clades while *Puntius sarana* and *Hypsibarbus wetmorei* remained as out-group (Figure 3, Figure S2, and Figure S3). This also revealed that *T. putitora*, *T. tor*, *T. khudree*, *T. sinensis*, *T. mussullah*, *T. mosal*, *T. malabaricus*, *T. douronensis*, *T. tambroides*, *N*. *hexagonolepis* and *N*. *stracheyi* clustered separately and are distinct species. All the samples of T_1_, T_2_ and T_3_ clustered in the same clade and nearest to *T. putitora*; N_1_ samples clustered with *N. hexagonolepis*; and the 6 N_2_ samples clustered in the same clade while the other N_2_ sample remained a bit distant. In addition, some database sequences reflected aberrant clustering like, 1) all sequences of *T. mosal mahanadicus* and *T. macrolepis* clustered with *T. putitora* and 2) a single sequence of *N. stracheyi* (accession number HM536922) clustered with *N. Hexagonolepis*.

## Discussion

**Figure 4:**
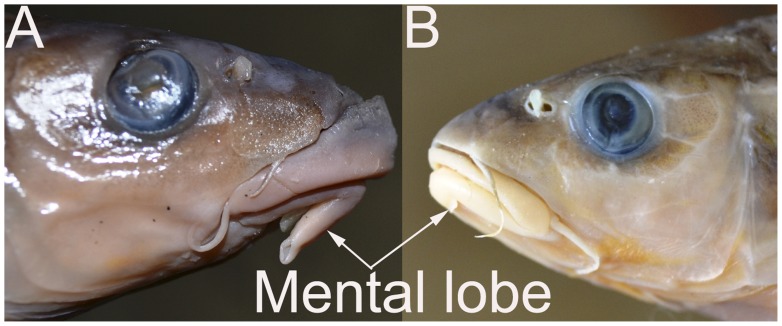
Illustrating the counter appearances of mouth in (A) *T. progeneius* and (B) *T. putitora*. Showing fleshy lips, a semicircular flap extending behind the upper lip, and a fleshy appendix extending from the lower lip up to the margin of maxilla (very long mental lobe) in (A), but absent in (B).

In this study, all the possible mahseer habitats across the Northeast India were surveyed. Altogether three morphologically distinct groups of mahseer within the genus *Tor* and two within *Neolissochilus* were identified from the study site. DNA barcoding analyses however recognized all the three groups belonging to a single species within the genus *Tor* and conspecific of *T. putitora*. The *T. putitora* is a widely distributed species and it has been reported to be exhibiting polymorphism in geographically isolated populations [42]. Among the study samples, T_3_ samples possessed long fleshy appendage to the lower lip (mental lobe) while the others lack this feature. This feature corresponds to the original description of *T. progeneius* where this particular feature was specially emphasized for nomenclature [18], [21]. This species had been also considered closely allied to *T. tor* in view of its lower lip character; consequently these two species have been synonymized very often [5], [19]. *T. progeneius* was however differently described after its original description probably due to lack of original holotype [3] and non-availability of fresh specimens [21]. It was identified to be distinct from *T. tor* due to length of head almost equal to depth of the body in the former vs. length of head considerably shorter than depth of the body in the latter [20]. Subsequently, based on archival specimens (Zoological survey of India, Kolkata; specimens’ catalogue details not mentioned), it was characterized to be having 8–10 rakers on the lower arm of first gill arch, tubercles on the cheek and lacking completely a mental lobe. Based on such characters this species was remarked to be doubtful to place in either in *Tor* or *Neolissochilus* [3]. According to one proposition, there are two types among the yellow finned mahseer: i) the lips are fleshy and the lower one is produced backwards into a long fleshy appendage, and ii) the lips are of normal type and the lower lip does not form an appendage [21]. Based on such descriptions, Hamilton’s *Cyprinus* (present *Tor*) *putitora* and *C. mosal* have been stated to be the same species and the nature of their lips was stated to be adaptive characters [5]. Besides, the description of a fan-shaped structure behind upper jaw in *T. progeneius* [21] was stated to be an abnormal formation based on archival specimens (Zoological Survey of India, Kolkata; specimens’ catalogue details not mentioned) [5]. In contrary, Menon (1992) [5] described *T. progeneius* to be possessing of 27–31 numbers of lateral line scales on the body. It seems that Menon (1992) was so influenced by this feature of *T. progeneius* that he used it as a taxonomic key to species. Secondly, in contrary to all previous descriptions except Rainboth (1985) [3], Menon (1992) noted the presence of cheek tubercles in *T. progeneius*. On the other hand, according to original description as well as the prevailing adoption of taxonomic character for this species indicate that the number of scales on the lateral line was never more than 26, and the extension of singular appendage from lower lip has been largely emphasized. This species has long been remained unreported, that might be due to the above mentioned morpho-taxonomic perplexity arising from vague and varied presentation of its specific characters incongruent to the original description [18]. All the T_3_ samples were observed to be possessing of maximum of 26 lateral line scales, 13–14 gill rakers, the slightly longer head than body depth and particularly the fleshy lips with long angular appendage to the lower jaw (long mental lobe) that is in contrast to the short mental lobe in both T_1_ and T_2_ samples (Figure 4). The different lower lip structure in T_3_ samples could be an adaptive [5], [21] or a sexually dimorphic feature [43]. Moreover, different geographical populations of *T. putitora* have been reported for significant Nuclear Organiser Region polymorphism [42] that indicates the possibility of the presence of a polymorphic form of this species in northeast India. Because, the collection site of T_3_ samples in the drainages of river Brahmaputra is phylogeographically poorly connected with the other Himalayan streams such as Ganga. Therefore, notwithstanding such noticeable differences in mouth structure, following DNA barcoding results, we conclude that *T. progeneius* is a synonymous species of *T. putitora*. This study contributed 10 replica barcode sequences in GenBank of *T. putitora.* In elsewhere, DNA barcoding approach has been successful in describing different nominal species in one [44]. This study would guide the conservationists to turn away the focus of conservation endeavor from *T. progeneius* to *T. putitora*.

The present study recognized two morphologically distinct groups of mahseer within the genus *Neolissochilus*. Among them, the N_1_ and N_2_ samples were identified to be *Neolissochilus hexagonolepis* and *N. hexastichus* respectively. DNA barcoding also differentiated both the species with considerable barcode gap and hence their identifications were confirmed. This study added in GenBank 3 replica barcode sequences of *N. hexagonolepis* and 7 new barcode sequences of *N. hexastichus*. The latter species has long been concealed since its first description in around 175 year back [18] due to lack of its morpho-taxonomic details and mis-identification with *T. tor*. The species *N. hexastichus* is though reported from other locations in the Salween basin [45] and Myanmar [46] but there are almost no biological data available on this species. Yet, it was first categorized into ‘Vulnerable’ [15] and subsequently to ‘Near Threatened’ status [16]. In this study, two species of mahseer, viz., *T. putitora* and *N. hexagonolepis* were found frequently in all the mahseer habitats in the study area. On the other hand, the species *N. hexastichus* was absent in all the surveyed habitats except a particular river (25.420 N 92.993 E) in the entire study area that raises a serious concern about the future sustainability of this species. Although this river also harbors the other two most common species of mahseer but we observed illegal harvest of fishes through destructive fishing in the river. Thus, the mahseer species in this river are assumed to have been threatened from anthropogenic activities that demands mass awareness. This study would provide benefit to generate life history parameters of *N. hexastichus* for its conservation standpoint and development of aquaculture package of practice for sustainable utilization. Therefore, this study suggests to initiate priority conservation of *N. hexastichus*.

One of the differentiating characters of two genera *Neolissochilus* and *Tor* is based on the presence of labial groove interrupted in the former and continuous in the latter. This generic character was found to be confusing because this difference was not evident in *N. hexastichus*. Therefore, we consider that interrupted labial groove would be confusing to treat as the generic character of *Neolissochilus*. On the other hand, the characteristic difference of the number of gill rakers on the first arm of gill arch was found to be a very pronounced generic character of the two genera that may be emphasized in genus categorization.

The NJ, ML and Bayesian cluster showed that the genera *Puntius* and *Hypsibarbus* remained as out-group with respect to the two genera *Tor* and *Neolissochilus* of mahseer. In another study the two genera of mahseer have been proposed to be in a distinct clade compared to other six different clades within the subfamily Cyprininae [47]. So, the grouping of mahseer in a separate tribe [34], [48] appeared justified, but, the particular tribe name is contentious. In the NJ phylogenetic analysis, some sequences, e.g., *T. macrolepis* (2 sequences) and *T. mosal mahanadicus* (3 sequences), though carried distinct names in the database but clustered cohesively with a popular species *T. putitora*. Such a wrong clustering of sequences may arise either due to misidentification or due to the occurrence of synonymous species, such as *T. macrolepis* has been stated to be a synonym of *T. putitora* [11], [17]. Besides, the two samples of *Neolissochilus stracheyi* did not cluster with each other and have been possibly misidentified in the database.

In the history of taxonomy, the dawn of DNA barcoding technique has sufficiently helped in troubleshooting of many species identification where morphological characters were overlooked or overemphasized [29]. Yet, the reference database is found to be lacking of information on many extant species of mahseer. Hence, development of both new barcodes and replica barcodes from wide spatial scale would be important to enrich the DNA barcode reference library. New barcodes are particularly essential to achieve the objective of DNA barcoding to complete the digital taxonomic guide of earth’s biota, while the replica barcodes from wide geographical ranges would substantiate the range distribution of the extant species.

## Supporting Information

Figure S1 **Scheme of measurement of morphometric variables on Fish.** (adopted from Jayaram (1999) [23].
(TIF)

Figure S2 **ML phylogeny.** The tagging of the sequences with red and black dots as well as black triangles follow the same description as given for NJ phylogenetic tree in Figure 3.
(TIF)

Figure S3 **Bayesian phylogeny.** The specimens’ GenBank accession number and species name are shown for each taxon. The sequences highlighted with red and blue colour correspond to the sequences developed in this study while blue coloured sequences alone correspond to the sequences of samples morphologically identified as *Tor progeneius,* but are found conspecific with *Tor putitora* in this study hence, marked as *Tor putitora.* The green coloured sequences correspond to the cases of abnormal clustering.
(TIF)

Table S1 **List of the studied species, GenBank Accession of the analyzed sequences and the geographical positions of the sample.**
(DOC)

Table S2 **Morphometric details of the studied species.**
(DOC)

Table S3 **Pairwise K2P divergence matrix between the sequences.**
(XLS)

Supporting Information S1 **Comparison of taxonomic descriptions based on morphology of** ***T. Progeneius*** **from time to time.**
(DOC)
