# Supplementary figures and images for: The Species Dilemma of Northeast Indian Mahseer (Actinopterygii: Cyprinidae): DNA Barcoding in Clarifying the Riddle
Source: PLoS One. 2013 Jan 16;8(1):e53704. doi: 10.1371/journal.pone.0053704 (PMC3547047; doi:10.1371/journal.pone.0053704)

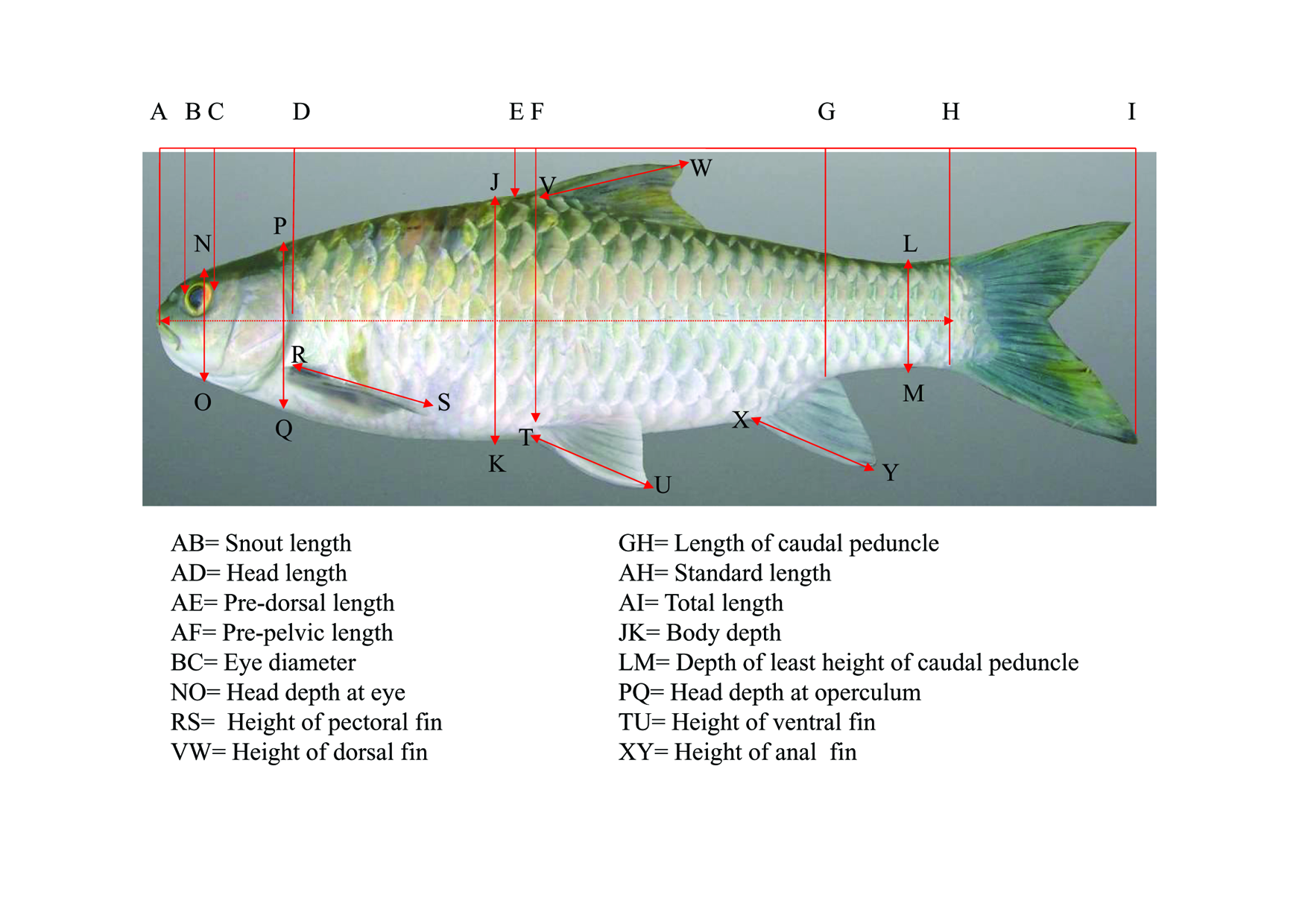

Supplement: Figure S1 — Scheme of measurement of morphometric variables on Fish. (adopted from Jayaram (1999) [23]. (TIF) [file pone.0053704.s001.tif]

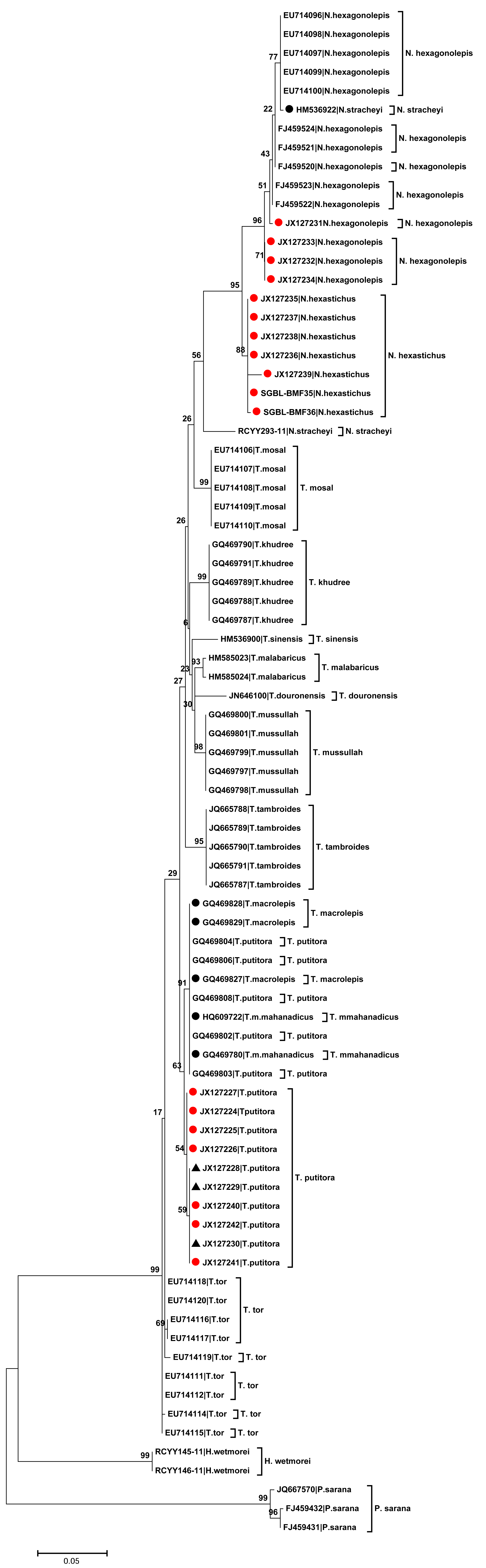

Supplement: Figure S2 — ML phylogeny. The tagging of the sequences with red and black dots as well as black triangles follow the same description as given for NJ phylogenetic tree in Figure 3. (TIF) [file pone.0053704.s002.tif]

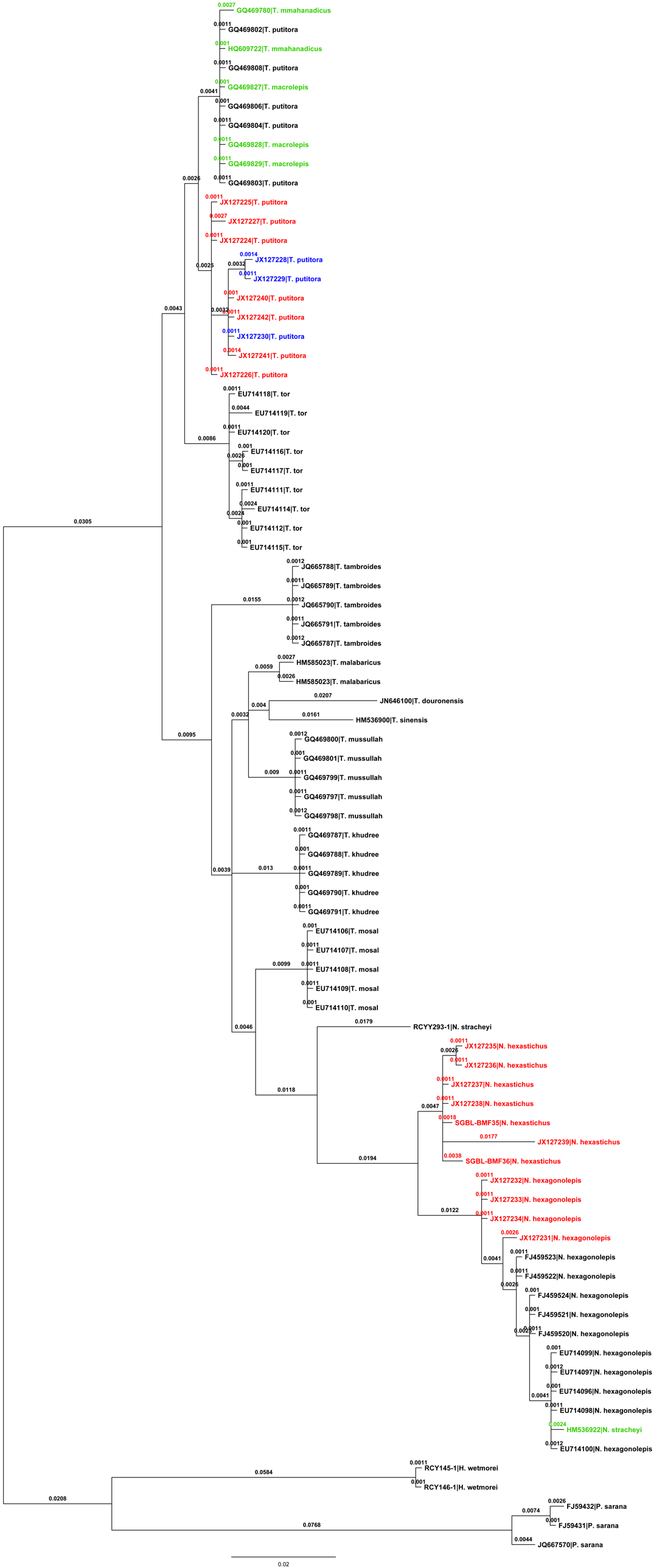

Supplement: Figure S3 — Bayesian phylogeny. The specimens’ GenBank accession number and species name are shown for each taxon. The sequences highlighted with red and blue colour correspond to the sequences developed in this study while blue coloured sequences alone correspond to the sequences of samples morphologically identified as Tor progeneius, but are found conspecific with Tor putitora in this study hence, marked as Tor putitora. The green coloured sequences correspond to the cases of abnormal clustering. (TIF) [file pone.0053704.s003.tif]
