# Supplementary material for: The Species Dilemma of Northeast Indian Mahseer (Actinopterygii: Cyprinidae): DNA Barcoding in Clarifying the Riddle
Source: PLoS One. 2013 Jan 16;8(1):e53704. doi: 10.1371/journal.pone.0053704 (PMC3547047; doi:10.1371/journal.pone.0053704)
Supplement: Table S1 — List of the studied species, GenBank Accession of the analyzed sequences and the geographical positions of the sample. (DOC) [file pone.0053704.s004.doc]

**Table S1.** List of species included in the study mentioning COI GenBank accession numbers of individuals analyzed under each species. The dataset includes 81 sequences of 16 species, among them 21 sequences were developed *denovo*, 55 sequences of Mahseer and 5 sequences as out-group of the study were acquired from NCBI GenBank.

| **Family** | **Studied species** | **COI GenBank**  **Accession No.** | **Geographical**  **Position** |
| --- | --- | --- | --- |
|  |  |  |  |
| *Cyprinidae* | 1. *Tor mosal* | EU714106-10 | N/A |
|  | 1. *Tor tor* | EU714111-12 | N/A |
|  |  | EU714114-20 | N/A |
|  | 1. *Tor mosal mahanadicus* | GQ469780 | N/A |
|  |  | HQ609722 | N/A |
|  | 1. *Tor khudree* | GQ469787-91 | N/A |
|  | 1. *Tor mussullah* | GQ469797-801 | N/A |
|  | 1. *Tor putitora* | GQ469802-04 | N/A |
|  |  | GQ469806 | N/A |
|  |  | GQ469808 | N/A |
|  |  | *JX127224 | 27.511 N 96.079 E |
|  |  | *JX127225 | 28.264 N 95.708 E |
|  |  | *JX127226 | 28.171 N 95.831 E |
|  |  | *JX127227 | 28.157 N 95.869 E |
|  |  | *JX127228 | 27.511 N 96.079 E |
|  |  | *JX127229 | 28.264 N 95.708 E |
|  |  | *JX127230 | 28.157 N 95.869 E |
|  |  | *JX127240-42 | 28.100 N 95.304 E |
|  | 1. *Tor macrolepis* | GQ469827-29 | N/A |
|  | 1. *Tor sinensis* | HM536900 | N/A |
|  | 1. *Tor malabaricus* | HM585023-24 | N/A |
|  | 1. *Tor douronensis* | JN646100 | 4.65 N 116.95 E |
|  | 1. *Tor tambroides* | JQ665787-91 | N/A |
|  | 1. *N. hexagonolepis* | FJ459520 | 25.38 N 91.52 E |
|  |  | FJ459521 | 25.38 N 91.52 E |
|  |  | FJ459522 | 25.38 N 91.52 E |
|  |  | FJ459523 | 25.38 N 91.52 E |
|  |  | FJ459524 | 25.38 N 91.52 E |
|  |  | *JX127231 | 27.511 N 96.079 E |
|  |  | *JX127232 | 28.147 N 95.917 E |
|  |  | *JX127233 | 28.264 N 95.708 E |
|  |  | *JX127234 | 28.157 N 95.869 E |
|  |  | EU714096-100 | N/A |
|  | 1. *N. stracheyi* | HM536922 | N/A |
|  |  | RCYY293 | N/A |
|  | 1. *N. hexastichus* | *JX127235-39 | 25.420 N 92.993 E |
|  |  | SGBL-BMF35-36 | 25.420 N 92.993 E |
|  | 1. *Puntius sarana* | JQ667570 | 22.948 N 81.077 E |
|  |  | FJ459431-32 | 24.57 N 88.06 E |
|  | 1. *Hypsibarbus wetmorei* | RCYY145-46 | N/A |

- ‘NA’ denotes not available; *sequences developed *denovo*
