# Supplementary material for: The Species Dilemma of Northeast Indian Mahseer (Actinopterygii: Cyprinidae): DNA Barcoding in Clarifying the Riddle
Source: PLoS One. 2013 Jan 16;8(1):e53704. doi: 10.1371/journal.pone.0053704 (PMC3547047; doi:10.1371/journal.pone.0053704)
Supplement: Table S2 — Morphometric details of the studied species. (DOC) [file pone.0053704.s005.doc]

**Table S2. Morphometric details of the fresh and previously identified samples as well as available paratypes of the studied species.** These data are used in PCA and sample codes represented in Table 1 with the corresponding sequence codes. Abbreviations of the variables code are detailed in the foot note.

| **Species name**  **↓**  **Sample**  **Codes ↓** | | ***Variables (values in percent of total body length)** | | | | | | | | | | | | | | | | | | | | | | | |
| --- | --- | --- | --- | --- | --- | --- | --- | --- | --- | --- | --- | --- | --- | --- | --- | --- | --- | --- | --- | --- | --- | --- | --- | --- | --- |
|  |  | SL | PrDL | PoDL | HtCF | HL | HtPF | HtDF | HtAF | HtDS | DP&V | LnCP | BDdf | HDop | HDe | BWdf | HWe | SnL | ED | LnLF | LHtCP | HtVF | DVF&AF | LnBDF | LnBAF |
| *N. hexastichus* | A | 72.85 | 39.90 | 47.59 | 25.08 | 21.69 | 16.04 | 22.73 | 16.46 | 18.55 | 16.97 | 9.71 | 24.66 | 19.06 | 13.36 | 11.95 | 11.41 | 7.48 | 5.21 | 1.54 | 9.93 | 16.07 | 15.70 | 11.66 | 5.96 |
|  | B | 74.34 | 40.95 | 53.21 | 23.88 | 22.03 | 15.92 | 24.29 | 16.83 | 17.99 | 16.07 | 8.13 | 24.44 | 18.38 | 13.75 | 9.65 | 11.14 | 6.85 | 6.03 | 1.65 | 9.70 | 15.76 | 14.90 | 12.69 | 5.14 |
|  | C | 74.01 | 38.87 | 49.76 | 25.73 | 20.64 | 15.84 | 22.97 | 15.99 | 17.68 | 17.42 | 11.86 | 23.17 | 16.82 | 13.05 | 9.04 | 11.27 | 6.60 | 4.71 | 1.79 | 9.93 | 14.85 | 16.13 | 12.38 | 5.78 |
|  | D | 73.52 | 38.42 | 52.58 | 27.02 | 20.56 | 16.18 | 24.30 | 16.39 | 18.31 | 15.64 | 10.34 | 22.21 | 18.34 | 13.04 | 10.41 | 10.95 | 6.77 | 4.66 | 1.49 | 9.28 | 14.41 | 15.67 | 13.08 | 5.97 |
|  | E | 72.38 | 38.40 | 52.09 | 27.01 | 20.41 | 15.11 | 23.66 | 16.88 | 18.38 | 16.23 | 9.86 | 21.64 | 18.04 | 13.11 | 10.69 | 10.49 | 6.85 | 5.04 | 1.67 | 9.10 | 14.51 | 15.79 | 12.53 | 5.55 |
|  | F | 74.59 | 38.30 | 50.22 | 26.07 | 21.26 | 15.41 | 24.24 | 15.81 | 18.66 | 17.56 | 10.68 | 24.41 | 19.06 | 13.68 | 10.79 | 10.24 | 7.25 | 5.00 | 1.77 | 9.90 | 15.16 | 17.47 | 12.10 | 5.46 |
| *N. hexagonolepis* | G | 78.22 | 38.22 | 51.49 | 21.97 | 20.06 | 15.23 | 18.60 | 14.19 | 15.02 | 21.05 | 12.31 | 22.20 | 18.01 | 13.40 | 11.87 | 11.62 | 7.34 | 4.17 | 0.00 | 9.45 | 14.02 | 20.06 | 10.96 | 5.33 |
|  | H | 75.65 | 37.87 | 51.33 | 23.66 | 19.16 | 14.76 | 21.24 | 14.39 | 18.81 | 20.40 | 13.23 | 20.17 | 17.95 | 11.96 | 10.36 | 9.11 | 5.82 | 3.93 | 0.00 | 8.59 | 13.58 | 20.44 | 11.16 | 5.93 |
|  | I | 78.45 | 37.28 | 51.48 | 21.55 | 19.93 | 15.87 | 20.84 | 14.13 | 18.91 | 20.60 | 13.97 | 22.07 | 17.67 | 12.46 | 11.81 | 9.83 | 6.37 | 4.40 | 0.00 | 9.01 | 13.20 | 19.81 | 11.30 | 5.79 |
|  | V** | 75.11 | 37.55 | 51.53 | 24.32 | 20.46 | 15.73 | 19.85 | 14.20 | 16.22 | 18.36 | 9.84 | 21.47 | 17.82 | 13.10 | 9.76 | 9.23 | 6.32 | 4.60 | 0.00 | 9.57 | 13.37 | 19.87 | 12.31 | 5.23 |
|  | W** | 76.64 | 38.22 | 51.76 | 24.48 | 20.73 | 10.14 | 18.13 | 13.20 | 17.02 | 22.22 | 11.09 | 22.45 | 18.57 | 13.36 | 9.72 | 10.24 | 6.15 | 4.87 | 0.00 | 9.36 | 12.85 | 18.36 | 12.00 | 5.83 |
|  | X** | 74.85 | 38.94 | 52.47 | 24.59 | 18.70 | 14.79 | 19.91 | 13.25 | 17.43 | 19.39 | 11.45 | 20.51 | 15.57 | 12.84 | 9.84 | 9.54 | 5.73 | 4.60 | 0.00 | 8.92 | 13.31 | 17.79 | 19.01 | 5.66 |
| *T. progeneius* | J | 77.26 | 41.30 | 48.71 | 23.62 | 24.33 | 15.48 | 20.90 | 13.75 | 17.96 | 17.50 | 10.73 | 18.42 | 15.88 | 12.44 | 8.91 | 9.21 | 9.09 | 4.56 | 5.62 | 8.71 | 13.22 | 16.62 | 9.80 | 4.59 |
|  | K | 78.19 | 42.21 | 49.17 | 21.78 | 23.92 | 14.82 | 20.43 | 13.97 | 15.94 | 16.81 | 12.06 | 18.18 | 15.26 | 10.88 | 8.98 | 8.40 | 8.62 | 4.29 | 4.82 | 8.31 | 12.78 | 16.24 | 10.15 | 4.91 |
|  | L | 76.65 | 40.85 | 48.90 | 22.99 | 24.92 | 14.16 | 21.65 | 15.92 | 17.34 | 16.35 | 12.40 | 18.46 | 16.12 | 12.42 | 8.76 | 9.40 | 8.79 | 5.22 | 5.10 | 8.93 | 13.11 | 15.34 | 10.21 | 4.78 |
|  | M | 77.14 | 42.30 | 49.37 | 22.18 | 24.30 | 14.51 | 21.07 | 15.41 | 18.14 | 16.76 | 11.58 | 19.78 | 15.04 | 11.92 | 9.93 | 9.88 | 8.78 | 4.63 | 4.29 | 8.35 | 13.89 | 15.94 | 10.92 | 5.63 |
|  | N | 78.10 | 40.07 | 48.14 | 22.42 | 24.96 | 14.58 | 19.33 | 13.98 | 16.60 | 18.65 | 11.10 | 17.72 | 14.65 | 10.43 | 9.23 | 8.20 | 7.91 | 4.55 | 5.91 | 8.01 | 12.73 | 16.45 | 10.40 | 5.05 |
| *T. putitora* | Aa** | 77.11 | 37.75 | 49.48 | 22.57 | 22.76 | 14.28 | 21.66 | 14.20 | 17.95 | 14.75 | 12.78 | 18.87 | 15.47 | 12.08 | 8.56 | 8.85 | 7.86 | 4.59 | 1.98 | 8.79 | 13.41 | 15.92 | 12.10 | 4.46 |
|  | O | 76.79 | 38.63 | 49.04 | 23.99 | 23.69 | 13.80 | 22.56 | 14.36 | 19.19 | 19.45 | 10.45 | 18.41 | 15.56 | 12.38 | 9.21 | 9.37 | 7.68 | 4.96 | 1.96 | 8.51 | 12.96 | 17.93 | 11.98 | 5.03 |
|  | P | 76.27 | 39.27 | 49.92 | 23.70 | 22.39 | 14.88 | 21.52 | 14.65 | 17.53 | 16.80 | 11.64 | 17.97 | 14.93 | 11.54 | 9.23 | 8.65 | 6.81 | 4.31 | 1.81 | 8.01 | 13.11 | 16.20 | 10.70 | 5.25 |
|  | Q | 76.83 | 39.85 | 50.20 | 24.71 | 23.56 | 14.94 | 22.80 | 15.09 | 18.73 | 15.65 | 11.19 | 18.12 | 14.67 | 11.69 | 8.63 | 8.84 | 7.33 | 4.58 | 1.99 | 8.33 | 13.02 | 16.82 | 11.87 | 5.82 |
|  | U | 77.35 | 39.51 | 49.24 | 22.61 | 22.46 | 13.66 | 21.10 | 14.63 | 17.41 | 17.15 | 10.66 | 17.93 | 16.17 | 11.41 | 9.20 | 9.38 | 7.24 | 5.10 | 1.83 | 8.12 | 12.70 | 16.38 | 11.49 | 5.45 |
| *T. putitora* | R | 78.57 | 40.85 | 50.64 | 21.43 | 22.36 | 13.74 | 19.06 | 14.12 | 16.15 | 19.31 | 12.13 | 18.95 | 16.40 | 11.08 | 8.57 | 8.64 | 7.46 | 3.90 | 1.47 | 8.25 | 12.60 | 18.51 | 10.81 | 5.68 |
|  | S | 79.49 | 40.35 | 48.87 | 21.80 | 22.80 | 14.32 | 19.77 | 14.41 | 16.63 | 21.17 | 12.14 | 19.28 | 16.03 | 11.53 | 8.97 | 8.26 | 7.29 | 4.29 | 1.72 | 7.94 | 12.28 | 18.52 | 10.70 | 5.96 |
|  | T | 78.67 | 40.20 | 50.04 | 22.33 | 22.77 | 14.93 | 19.69 | 15.03 | 16.46 | 19.95 | 12.37 | 19.66 | 16.65 | 11.83 | 9.52 | 7.93 | 7.29 | 3.98 | 1.56 | 8.29 | 13.64 | 19.99 | 10.31 | 5.74 |
|  | Y** | 75.61 | 38.74 | 49.70 | 23.09 | 22.20 | 14.63 | 19.66 | 13.76 | 16.09 | 19.67 | 12.06 | 17.38 | 15.51 | 11.25 | 10.09 | 8.29 | 7.18 | 3.68 | 1.55 | 7.88 | 13.23 | 18.61 | 11.68 | 5.39 |
|  | Z** | 78.88 | 41.27 | 49.58 | 22.58 | 22.54 | 14.07 | 20.34 | 14.20 | 16.82 | 19.53 | 16.07 | 18.43 | 15.41 | 12.09 | 10.32 | 9.94 | 7.57 | 4.12 | 1.90 | 8.62 | 12.51 | 19.16 | 10.20 | 5.34 |
|  | Ab** | 77.71 | 40.31 | 48.99 | 21.51 | 23.18 | 14.37 | 22.46 | 12.66 | 16.86 | 19.07 | 12.27 | 18.93 | 15.95 | 12.30 | 8.72 | 9.03 | 8.53 | 4.18 | 1.07 | 8.66 | 12.66 | 17.61 | 10.07 | 5.44 |

*Abbreviations are: SL=Standard Length, PrDL=Pre-dorsal Length, PoDL=Post-dorsal Length, HtCF=Height of Caudal Fin, HL=Head Length, HtPF=Height of Pectoral Fin, HtDF=Height of Dorsal Fin, HtAF=Height of Anal Fin, HtDS=Height of Dorsal Spine, DP&V=Distance between Pectoral and Ventral, LnCP=Length of Caudal Peduncle, BDdf=Body Depth at dorsal fin origin, Hdop=Head Depth at operculum, Hde=Head Depth at posterior edge of eye, Hwe=Head Width at eye, SnL=Snout Length, ED=Eye Diameter, LnLF=Length of Labial Flap, LHtCP=Least Height of Caudal Peduncle, HtVF=Height of Ventral Fin, LnBDF=Length of base of Dorsal Fin, LnBAF=Length of base Anal Fin

**paratypes from museum
