# Supplementary material for: The Species Dilemma of Northeast Indian Mahseer (Actinopterygii: Cyprinidae): DNA Barcoding in Clarifying the Riddle
Source: PLoS One. 2013 Jan 16;8(1):e53704. doi: 10.1371/journal.pone.0053704 (PMC3547047; doi:10.1371/journal.pone.0053704)
Supplement: Supporting Information S1 — Comparison of taxonomic descriptions based on morphology of T. Progeneius from time to time. (DOC) [file pone.0053704.s007.doc]

**Additional information**

**COMPARISON OF TAXONOMIC DESCRIPTIONS BASED ON MORPHOLOGY OF *T. progeneius* FROM TIME TO TIME**

**Prelude**

In the history of taxonomy and inventory of Indian freshwater fishes, Hamilton (1822) considered many species (44) under the **genus *Cyprinus*** those are now classified into several genera. He considered three species in one group having strong resemblances, in his words, being very large fishes, with enormous size of scales, very strong, well formed, handsome fishes and referred as **mahseer**. He described three species, *C. putitora* having jaws protruding in opening and upper jaw longest, *C. tor* having a protuberance between nostrils, and *C. mosal* having small tubercle between nostrils as in the last species but less strongly marked. He did not cover the fishes from the **Northeast of India** but up to Bengal which were discriminately fulfilled by McClelland (1839) with the record of five species of mahseer under the **old named** **genus *Barbus*** Cuvier; among them, according to **modern nomenclature**, three are classified under the **genus *Tor*** and two under **genus** ***Neolissochilus***. A brief comparison of the morphological characters of *T. progeneius* described by different workers is given below to reveal the existing dispute in taxonomy of this species. The descriptions by earlier workers were large descriptive that pose difficulty in presenting the comparison in tabular or pictorial form. However, the important issues are underlined and a brief remark is given at the end. The reference number as used in the MS text is uniformly used in this comparison to maintain link with the original text.

**Comparison**

**McClelland (1839)** originally described the ***Barbus progeneius*** having length of the head to that of the body as one to three, 26 scales along the lateral line and 6 in an oblique row from the base of the ventrals to the dorsum. Mouth is narrow and small, and from the lower lip a fleshy appendix is extended by which it is distinguished from the neighbouring species [18].

**Jerdon (1849)** considered ***B. progeneius*** as synonymous with *B. hamiltoni* (Gray, 1834) which he described as head to the whole body as 1 to 3½, a fleshy projection on both upper and lower lips. Body compressed, its height is to total length as 1 to 3½, 26 scales along the lateral line in 6 rows, green above, cheeks golden, silvery beneath, fins tinged red.

Hora (1936) discussed that Gray’s *Tor hamiltoni* is the *Tor* mahseer of northern India but in identifying the peninsular form *Barbus khudree* with McClelland’s *B. progeneius* Jerdon seems to have been influenced by the character of the enlarged lips which is probably common in all species of mahseer. *T. hamiltoni* is now considered as junior synonym of *T. tor*.

**Day (1868)** considered *Barbus mosal* and *B. putitora* of Hamilton, and *B. macrocephalus* of McClelland to be one species under the name *B. mosal*; and *B. tor* and *B. progeneius* to be a distinct species under the name *B. tor*. The difference between these two forms was stated to consist in the latter having a pointed snout, the lower jaw being the shortest, mouth somewhat deeply cleft, lips thick and cartilaginous with protruding lobes [19].

**Gunther (1868)** considered *B. progeneius* as junior synonym of *B. mosal*. He also considered other synonyms of *B. mosal* as *B. hamiltoni, B. megalepis* and *B. khudree*. He considered *B. macrocephalus* of McClelland and *B. macrolepis* of Heckel as distinct species.

**Beavan (1877)** included seven varieties of mahseer under the name of *B. mosal*. He stated that all varieties show no sufficient differences by which to identify each variety separately with any amount of certainty as they merge one into another. The principal difference was stated in the shape of the head. Further he stated that as two of these varieties (viz. *B. progeneius* and *B. macrocephalus*) were known to be natives of Assam by different names there is reason to believe that they may really be distinct species.

**Hora (1941)** [21] stated that the material in the collection of the Zoological Survey of India (ZSI) Calcutta referred to as ***Barbus* (*Tor*) *progeneius*** was inadequate and was not sufficient for the determination of the precise specific limits of the species. He focused on the descriptions of McClelland that the *B. progeneius* has a prominent chin or long beard. In allusion to the singular appendage to the lower jaw of the species by which it may be easily recognized. He compared the illustrations of Hamilton’s *Cyprinus tor* and McClelland’s *B. progeneius* and stated that the two can not represent the same species. In *C. tor*, the head is more pointed and the body is considerably deeper and more pronounced along the ventral surface, while in *B. progeneius* the head is evenly pointed and is more or less equal to the depth of the body which is slender and graceful. He further stated that the presence of a labial fold is not a very sound taxonomic character as the development of this structure probably depends on certain undetermined environmental factors. He elicited further information and proposed to regard them as distinct species.

Hora recorded some descriptions of *B. progeneius* based on stuffed specimens. The head was sharpish in front, its length was contained from 4.5 to 4.9 times in the total length and from 3.5 to 3.8 times in the standard length. The depth of the body was more or less equal to the length of the head and the least height of caudal peduncle was contained from 1.4 to 1.8 times in its length. He recorded that behind the upper lip there was a rounded, fan shaped structure which in form and extend was quite different from the hypertrophied lips in *B. putitora* and *B. tor*. Further he mentioned that as such structure has not been observed in any other specie so far, it may be an abnormal condition

A number of specimens from the Naga Hills (Assam) have been deposited in ZSI Calcutta and referred to as *B. progeneius* by Hora (1936) but he remarked that if it proves to be a synonym of *B. mosal* then the fish will have a much wider range of distribution. Measurements of four such specimens from Naga hills recorded by Hora (1941) are presented below.

**Sen and Jayaram (1982)** [20] remarked that *T. progeneius* had been regarded as a synonym of *T. tor* or other closely allied *Tor* species due to its lower lip character which is produced into a median fleshy lobe in the adult form. They described the species with the diagnostic characters like head is long and much compressed, fins short, large scales, mouth narrow and small, from the lower lip a fleshy appendix is extended by which it is distinguished from the neighboring species, dorsal spine less developed, length of head almost equal to depth of body, tip of snout fleshy and produced into semicircular flap, upper lip fleshy but not projected, continuous labial fold, a black streak behind the gill opening in younger forms.

**Rainboth (1985)** [3] discussed the relationship between the genera *Tor* and *Neolissochilus* and stated that gill raker counts can distinguish them well. He studied the museum specimens in ZSI Calcutta and found that *Neolissochilus* have 6 to 9 gill rakers and all *Tor* except *T. progeneius* have 10 to 16 rakers. *T. progeneius* has 8 to 10 rakers on the lower arm of the first arch and appears to be an intermediate between *Tor* and *Neolissochilus*. Further, he recorded that *T. progeneius* is the only gengetic *Tor* with cheek tubercles and the only *Tor* anywhere which may occasionally lack completely a median lobe on the lower lip.

**Menon (1992)** [5] described the diagnostic features of the species, based on the specimens from Barak river in ZSI Calcutta, as a graceful streamlined mahser with the length of head almost equal to depth of body, and scales along lateral line 27 to 31 rows. Lips fleshy, smooth edged, continuous at the angles of mouth with uninterrupted fold or groove along lower jaw, lower lip with a median lobe. Dorsal fin almost in the middle of body, the dorsal spine weak, snout pointed and the lateral sides of snout covered with a series of tubercles. He recorded the relative length and structure of different fins. Further, he stated that the fan shaped structure behind upper jaw (a semi-circular flap at tip of snout) described by earlier workers is an abnormal formation.

**Talwar and Jhingran (1991)** [4] provides the taxonomic key to species based on previous descriptions and recognized 7 species within the genus *Tor* including *T. chelynoides*. They describe the species of *T. progeneius* having depth of body 3.5 to 3.8 times in standard length, length of head equal to or shorter than body depth, median fleshy lobe of lower lip occasionally lacking, behind upper lip a fan shaped flap present, gill rakers 8 to 10 on the lower arm of first arch, lateral line with 24 to 27 scales, snout smooth but cheeks with tubercles. In the keys, *T. khudree* was characterized as having a patch of indistinct tubercles on snout and *T. mussullah* having both snout and cheeks covered with rows of indistinct tubercles.

**Jayaram (1999)** [23] provides the taxonomic keys to species and recognized 7 species including *T. neilli* but excluding *T. chelynoides*. He describes *T. progeneius* having a fan shaped rounded structure behind upper lip, gill rakers 8 to 10 on lower arm of first gill arch and length of head considerably shorter or more or less equal to body depth.

**Remarks**

This comparison clearly indicated disputes in morphological descriptions of *T. progeneius*. McClelland (1839) described this species emphasizing on the fleshy appendix extended from the lower lip and presence of 26 scales along lateral line. Jerdon (1849) considered *B. progeneius* as synonym of *B. hamiltoni* having a fleshy projection on both upper and lower lips and 26 scales on lateral line. Day (1868) considered *B. tor* and *B. progeneius* as single having a pointed snout, shorter lower jaw and lips with protruding lobes. Gunther (1868) considered *B. progeneius* a junior synonym of *B. mosal*. Beavan (1877) although considered *B. mosal* as the only mahseer species but agreed to believe *B. progeneius* could be a distinct species. Hora (1941) tried to identify *B. progeneius* but failed due to inadequate material in ZSI Calcutta. He agreed with the original descriptions and stated that *Cyprinus tor* and *B. progeneius* can not be the same species as the former has deeper body and the latter has slender body. He further stated that the presence of a labial fold is not a very sound taxonomic character. He had submitted in ZSI a few specimens collected from Naga Hills in Assam and considered them to be *B. progeneius*, but remarked that they could be synonym of *B. mosal*. Sen and Jayaram (1982) claimed *B. progeneius* to be a distinct species having a fleshy appendix extended from lower lip, length of head equal to depth of body. They stated a few new characters that tip of snout is fleshy and produced into a semicircular flap and a black streak behind the gill opening in younger forms. Rainboth (1985) also stated a few characters based on the specimens at ZSI Calcutta that *T. progeneius* has 8 to 10 rakers on the lower arm of first arch, tubercles on cheek and may occasionally lack completely a median lobe on the lower lip. Menon (1992) further described a few characters based on ZSI specimens that body of *T. progeneius* covers with 27 to 31 scales along lateral line, and lateral sides of snout cover with a series of tubercles but he disagreed on the presence of fanshaped structure behind upper jaw.

Thus, it appears that *T. progeneius* has been described severally and many often questions raised against the validity of the species. Nevertheless, the recurrent descriptions have missed the key characters those were emphasized in original description. This indicates that the descriptions of *T. progeneius* by different workers might had based on different specimens those might represent distinct species or morphs of any species other than those already described.
